# Named Data Networking for Genomics Data Management and Integrated Workflows

**DOI:** 10.3389/fdata.2021.582468

**Published:** 2021-02-15

**Authors:** Cameron Ogle, David Reddick, Coleman McKnight, Tyler Biggs, Rini Pauly, Stephen P. Ficklin, F. Alex Feltus, Susmit Shannigrahi

**Affiliations:** ^1^ School of Computing, Clemson University, Clemson, SC, United States; ^2^ Department of Computer Science, Tennessee Tech University, Cookeville, TN, United States; ^3^ Department of Genetics and Biochemistry, Clemson University, Clemson, SC, United States; ^4^ Department of Horticulture, Washington State University, Pullman, WA, United States; ^5^ Biomedical Data Science and Informatics Program, Clemson, SC, United States; ^6^ Center for Human Genetics, Clemson University, Greenwood, SC, United States

**Keywords:** genomics data, genomics workflows, large science data, cloud computing, named data networking

## Abstract

Advanced imaging and DNA sequencing technologies now enable the diverse biology community to routinely generate and analyze terabytes of high resolution biological data. The community is rapidly heading toward the petascale in single investigator laboratory settings. As evidence, the single NCBI SRA central DNA sequence repository contains over 45 petabytes of biological data. Given the geometric growth of this and other genomics repositories, an exabyte of mineable biological data is imminent. The challenges of effectively utilizing these datasets are enormous as they are not only large in the size but also stored in geographically distributed repositories in various repositories such as National Center for Biotechnology Information (NCBI), DNA Data Bank of Japan (DDBJ), European Bioinformatics Institute (EBI), and NASA’s GeneLab. In this work, we first systematically point out the data-management challenges of the genomics community. We then introduce Named Data Networking (NDN), a novel but well-researched Internet architecture, is capable of solving these challenges at the network layer. NDN performs all operations such as forwarding requests to data sources, content discovery, access, and retrieval using content names (that are similar to traditional filenames or filepaths) and eliminates the need for a location layer (the IP address) for data management. Utilizing NDN for genomics workflows simplifies data discovery, speeds up data retrieval using in-network caching of popular datasets, and allows the community to create infrastructure that supports operations such as creating federation of content repositories, retrieval from multiple sources, remote data subsetting, and others. Named based operations also streamlines deployment and integration of workflows with various cloud platforms. Our contributions in this work are as follows 1) we enumerate the cyberinfrastructure challenges of the genomics community that NDN can alleviate, and 2) we describe our efforts in applying NDN for a contemporary genomics workflow (GEMmaker) and quantify the improvements. The preliminary evaluation shows a sixfold speed up in data insertion into the workflow. 3) As a pilot, we have used an NDN naming scheme (agreed upon by the community and discussed in Section 4) to publish data from broadly used data repositories including the NCBI SRA. We have loaded the NDN testbed with these pre-processed genomes that can be accessed over NDN and used by anyone interested in those datasets. Finally, we discuss our continued effort in integrating NDN with cloud computing platforms, such as the Pacific Research Platform (PRP). The reader should note that the goal of this paper is to introduce NDN to the genomics community and discuss NDN’s properties that can benefit the genomics community. We do not present an extensive performance evaluation of NDN—we are working on extending and evaluating our pilot deployment and will present systematic results in a future work.

## 1 Introduction

Scientific communities are entering a new era of exploration and discovery in many fields driven by high-density data accumulation. A few examples are Climate Science (Cinquini et al., 2014), High Energy Particle physics (HEP) (Aad et al., 2008), Astrophysics (Dewdney et al., 2009; LSST Dark Energy Science Collaboration, 2012), Genomics (Sayers et al., 2020), Seismology (Tsuchiya et al., 2012), Biomedical research (Luo et al., 2016), just to name a few. Often referred to as “data-intensive” science, these communities utilize and generate very large volumes of data, often reaching into Petabytes (Shannigrahi et al., 2018b) and soon projected to reach into Exabytes.

Data-intensive science has created radically new opportunities. Take for example high-throughput DNA Sequencing (HTDS). Until the very recent years, HTDS was slow, expensive, and only a few institutes were capable of performing it at scale (McCombie et al., 2019). With the advances in supercomputers, specialized DNA sequencers, and better bioinformatics algorithms, the effectiveness and cost of sequencing has dropped considerably and continues to drop. For example, sequencing the first reference human genome cost around $2.7 Billion over 15 years, and currently, it costs under $1,000 to resequence a human genome (Genome.gov, 2020). With commercial incentives, several companies are offering fragmented genome re-sequencing under $100, performed in only a few days. This massive drop in cost and improvement in speed supports more advanced scientific discovery. For example, earlier scientists could only test their hypothesis on a small number of genomes or gene expression conditions within or between species. With more publicly available datasets (Sayers et al., 2020), scientists can test their hypothesis against a larger number of genomes, potentially enabling them to identify rare mutations, precisely classify diseases based on a specific patient, and thus more accurately treat the disease (Lowy-Gallego et al., 2019).

While the growth of DNA sequencing is very encouraging, it has also created difficulty in genomics data management. For example, the National Center for Biotechnology Information’s (NCBI) Sequence Read Archive (SRA) database hosts 42 Petabytes of publicly accessible DNA sequence data (NCBI, 2019). Scientists desiring to use public data must discover (or locate) the data and move it from globally distributed sites to on-premize clusters and distributed computing platforms, including public and commercial clouds. Public repositories such as the NCBI SRA contain a subset of all available genomics data (Stephens et al., 2015). Similar repositories are hosted by NASA, NIH, and other organizations. Even though these datasets are highly curated, each public repository uses their own standards for data naming, retrieval, and discovery that makes locating and utilizing these datasets difficult.

Moreover, data management problems require the community to build and the scientists to spend time learning complex infrastructures (e.g., cloud platforms, grids) and creating tools, scripts, and workflows that can (semi)- automate their research. The current trend of moving from localized institutional storage and computing to an on-demand cloud computing model adds another layer of complexity to the workflows. The next generation of scientific breakthroughs may require massive data, thus, our ability to manage, distribute, and utilize extreme-scale datasets and securely integrate them with computational platforms may dictate our success (or failure) in future scientific research.

Our experience in designing and deploying protocols for big-science (Olschanowsky et al., 2014; Fan et al., 2015; Shannigrahi et al., 2015; Shannigrahi et al., 2017; Shannigrahi et al., 2018a; Shannigrahi et al., 2018b) suggests that 1) using hierarchical and community-developed names for storing, discovering, and accessing data can dramatically simplify scientific data management systems; 2) the network is the ideal place for integrating domain workflows with distributed services. In this work, we propose a named ecosystem over an evolving but well-researched future Internet architecture, Named Data Networking (NDN). NDN utilizes content names for all data management operations such as content addressing, content discovery, and retrieval. Utilizing content names for all network operations massively simplifying data management infrastructure. Users simply ask for the content by name (one such name might look like “/ncbi/homo/sapiens/hg38”) and the network delivers the content to the user.

Using content names that are understood by the end-user over an NDN network provides multiple advantages: natural caching of popular content near the users, unified access mechanisms, and location-agnostic publication of data and services. For example, a dataset properly named can be downloaded by, for example, NCBI or GeneLab at NASA, whichever is closer to the researcher. Additionally, the derived data (results, annotations, publications) are easily publishable into the network (possibly after vetting and quality control by NCBI or NASA) and immediately discoverable if appropriate naming conventions are agreed upon and followed. Finally, NDN shifts the trust to content itself; each piece of content is cryptographically signed by the data producer and verifiable by anyone for provenance.

The following sections are organized as follows. We first introduce NDN and the architectural constructs that make it attractive for the genomics community. We then discuss the data management and cyberinfrastructure challenges faced by the genomics community and how NDN can help alleviate them. We then present our pilot study applying NDN to a contemporary genomics workflow GEMmaker (Hadish et al., 2020) and evaluate the integration. Finally, we discuss future research directions and an integration roadmap with cloud computing services.

## 2 Named Data Networking

**FIGURE 1:**
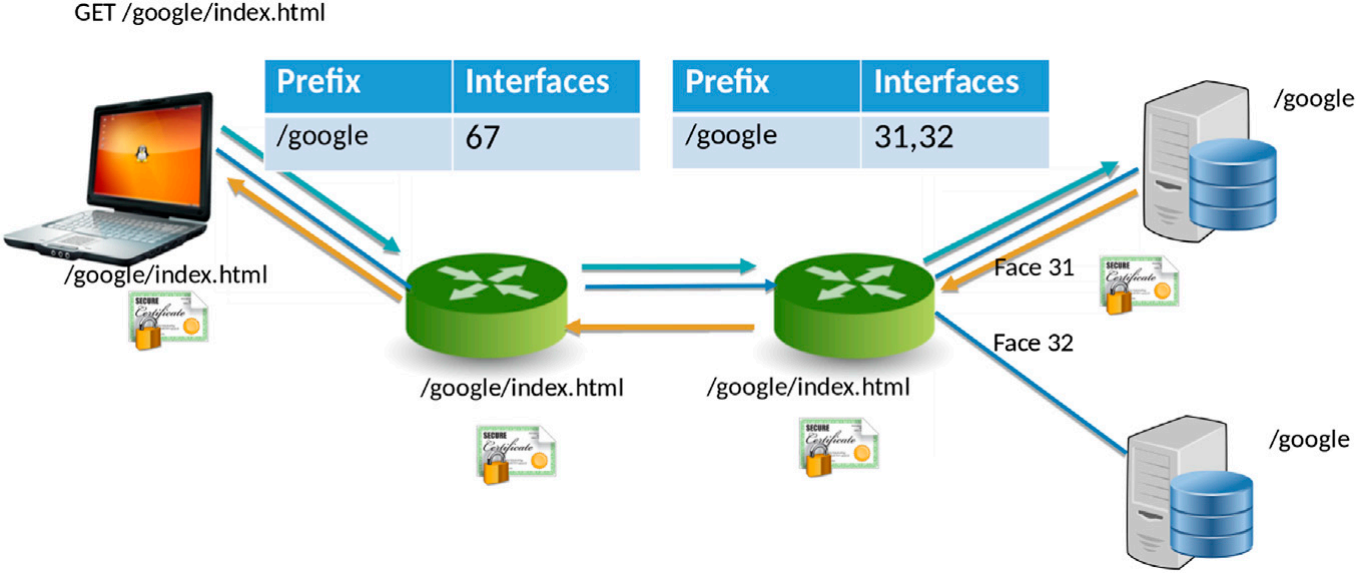
NDN Forwarding. The two servers on the right announce a namespace (/google) for the data they serve. The routers make a note of this incoming announcement, When the laptops ask for /google/index.html, the routers forward the requests on the appropriate interfaces (31, 32, or both, depending on configuration). Data follows the reverse path. (Zhang et al., 2014).

NDN (Zhang et al., 2014) is a new networking paradigm that adopts a drastically different communication model than that current IP model. In NDN, data is accessed by content names (e.g., “/Human/DNA/Genome/hg38”) rather than through the host where it resides (e.g., ftp://ftp.ncbi.nlm.nih.gov/refseq/H_sapiens/annotation/GRCh38_latest/refseq_identifiers/GRCh38_latest_genomic.fna.gz). Naming the data allows the network to participate in operations that were not feasible before. Specifically, the network can take part in discovering and local caching of the data, merging similar requests, retrieval from multiple distributed data sources, and more. In NDN, the communication primitive is straightforward (Figures 1A consumer asks for the content by content name (an “Interest” in NDN terminology), and the network forwards the request toward the publisher.

For communication, NDN uses two types of packets, *Interest* and *Data*. The content consumer initiates communication in NDN. To retrieve data, a consumer sends out an Interest packet into the network, which carries a name that identifies the desired data. One such content name (similar to a resource identifier) might be “/google/index.html”. A network router maintains a name-based forwarding table (FIB) (Figure 1). The router remembers the interface from which the request arrives, and then forwards the Interest packet by looking up the name in its FIB. FIBs are populated using a name-based routing protocol such as Named-data Link State Routing Protocol (NLSR) (Hoque et al., 2013).

NDN routes and forwards packets based on content names (Afanasyev et al., 2018), which eliminates various problems that addresses pose in the IP architecture such as address space exhaustion, Network Address Translation (NAT) traversal, mobility, and address management. In NDN, routers perform component-wize longest prefix match of the Interest name the FIB. Routing in NDN is similar to IP routing. Instead of announcing IP prefixes, an NDN router announces name prefixes that it is willing to serve (e.g., “/google”). The announcement is propagated through the network and eventually populates the FIB of every router. Routers match incoming Interests against the FIB using longest prefix match. For example, “/google/videos/movie1. mpg” might match “/google” or “/google/video”. Though an unbounded namespace raises the question of how to maintain control over the routing table sizes and whether looking up variable-length, hierarchical names can be done at line rate, previous works have shown that it is indeed possible to forward packets at 100 Gbps or more (So et al., 2013; Khoussi et al., 2019).

When the Interest reaches a node or router with the requested data, it packages the content under the same name (i.e., the request name), signs it with the producer’s signature, and returns it. For example, a request for “/google/index.html” brings back data under the same name “/google/index.html” that contains a payload with the actual data and the data producer’s (i.e., Google) signature. This Data packet follows the reverse path taken by the Interest. Note that Interest or Data packets do not carry any host information or IP addresses—they are simply forwarded based on names (for Interest packets) or state in the routers (for Data packets). Since every NDN Data packet is signed, the router can store it locally in a cache to satisfy future requests.

### 2.1 Hierarchical Naming

There is no restriction on how content is named in NDN except 1) they must be human-readable and hierarchical 2) and globally unique. The scientific communities develop the naming schemes as they see fit, and the uniqueness of names can be ensured by name registrars (similar to existing DNS Registrars).

The NDN design assumes hierarchically structured names, e.g., a genome sequence published by NCBI may have the name “/NCBI/Human/DNA/Genome/hg38”, where “/” indicates a separator between name components. The whole sequence may not fit in a single Data packet, so the segments (or chunks) of the sequence will have the names “/NCBI/Human/DNA/Genome/hg38/{1..n}“. Data that is routed and retrieved globally must have a globally unique name. This is achieved by creating a hierarchy of naming components, just like Domain Name System (DNS). In the example above, all sequences under NCBI will potentially reside under “/NCBI”; “/NCBI” is the name prefix that will be announced into the network. This hierarchical structure of names is useful both for applications and the network. For applications, it provides an opportunity to create structured, organized names. On the other hand, the network does not need to know all the possible content names, only a prefix, e.g., “/NCBI” is sufficient for forwarding.

### 2.2 Data-Centric Security

In NDN, security is built into the content. Each piece of data is signed by the data producer and is carried with the content. Data signatures are mandatory; on receiving the data, applications can decide if they trust the publisher or not. The signature, coupled with data publisher information, enables the determination of data provenance. NDN’s data-centric security helps establish data provenance, e.g., users can verify content with names that begin with “/NCBI” is digitally signed by NCBI’s key.

NDN’s data-centric security decouples content from its original publisher and enables in-network caching; it is no longer critical where the data comes from since the client can verify the authenticity of the data. Unsigned data is rejected either in the network or at the receiving client. The receiver can get content from anyone, such as a repository, a router cache, or a neighbor, as well as the original publisher and verify that the data is authentic.

### 2.3 In-Network Caching

Automatic in-network caching is enabled by naming data because a router can cache data packets in its content store to satisfy future requests. Unlike today’s Internet, NDN routers can reuse the cached data packets since they have persistent names and the producer’s signature. The cache (or Content Store) is an in-memory buffer that keeps packets temporarily for future requests. Data such as reference genomes can benefit from caching since caching the content near the user speeds up content delivery and reduces the load on the data servers. In addition to the CS, NDN supports persistent, disk-based repositories (repos) (Chen et al., 2014). These storage devices can support caching at a larger scale and CDN-like functionality without additional application-layer engineering.

In our previous work with Climate Science and High Energy physics community, we saw that even though scientific data is large, a strong locality of reference exists. We found that for climate data even a 1 GB cache in the network speeds up data distribution significantly (Shannigrahi et al., 2017). We observe similar patterns in the genomics community where some of the reference genomes are very popular. These caches do not have to be at the core of the network. We anticipate most of the benefits will come from caching at the edge. For example, a large cache provisioned at the network gateway of a lab will benefit the scientists at that lab. In this case, the lab will provision and maintain their caches. If data is popular across many organizations, it is in the operators best interest to cache the data at the core since this will reduce latency and network traffic. Given that storage price has gone down significantly (a 8 TB or 8000 GB hard-drive costs around $150, at the time of writing this paper), it does not significantly add to the operating costs of the labs. Additionally, new routers and switches are increasingly being shipped with storage, reducing the need for additional capital expenditure. Additionally, caching and cache maintenance is automated in NDN (it follows content popularity) eliminating the need to configure and maintain such storage.

Having introduced NDN in this section, we now enumerate the genomics data management problems and how NDN can solve them in the following section.

## 3 Genomics Cyberinfrastructure Challenges and Solutions Using NDN

The genomics community has made astronomical progress in recent decades. However, this progress has not been without challenges. A core challenge, like many other science domains, is data volume. Due to the low-cost sequencing instruments, the genomics community is rapidly approaching petascale data production at sequencing facilities housed in universities, research, and commercial centers. For example, the SRA repository at NCBI in Maryland, United States contains over 45 petabytes of high-throughput DNA sequence data—there are other similar genomic data repositories around the world (DDBJ, 2019; EBI, 2020). These data are complemented with metadata (though not always present or complete) representing evolutionary relationships, biological sample sources, measurement techniques, and biological conditions (NCBI, 2019).

Furthermore, while a large amount of data is accessible from large repositories such as the NCBI repository, a significant amount of genomics data resides in thousands of institutional repositories (Lathe et al., 2008; Dankar et al., 2018; National Genomics Data Center Members and Partners, 2019). The current (preferred) way to publish data is to upload it to a central repository, e.g., NCBI, which is time-consuming and often requires effort from the scientists. The massively distributed nature of the data makes the genomics community unique. In other scientific communities, such as high-energy physics (HEP), climate, and astronomy, only a few large scale repositories serve most of the data (Group and for Nuclear Research, 1991). For example, the LHC produces most of the data for the HEP community at CERN, the telescopes (such as LSST and to-be-built SKA) produces most of the data for astrophysics, and the supercomputers at various national labs produce climate simulation outputs (Taylor and Doutriaux 2010).

Modern genomic data comes in the form of 1) reference genomes with coordinate-based annotation files, 2) “dynamic” measurements of genome output (e.g., RNA-seq, CHIP-seq), and 3) individual genome resequencing data. Reference genomes are used by many researchers across the world that can benefit from efficient data delivery mechanisms. The dynamic functional genomics and resequencing genomics datasets are often larger in size and of more focused use. All data is typically retrieved using various contemporary technologies such as sneakernet (Munson and Simu, 2011), SCP/FTP, Aspera (ASPERA, 2019), Globus (glo, 2020), and iRODS (Rajasekar et al., 2010) (Chiang et al., 2011). While reference data is often easier to locate and download, the dynamic and resequencing datasets often are not since they are strewn over geographically distributed institutional repositories. Locating and retrieving data are not the only problems that the genomics community face—the rest of the section enumerates the cyberinfrastructure requirements of the genomics community, encountered problems due to the current point-to-point, TCP/IP based model of the Internet, and how NDN can solve these.

### 3.1 Problem-Massive Storage

The genomics community is producing more data than it is currently feasible to store locally (Stephens et al., 2015). This phenomenon will accelerate as modern field-based or hand-held sequencers become more prevalent in individual research labs and commercial sequencing providers. Increasingly, valuable data is at risk of being lost, potentially forever. While the community must invest in storage capacity, the existing storage strategies need to be optimized, such as deduplication of popular datasets (e.g., the reference genomes). Moreover, popular datasets that are often reused must be available quickly and reliably to reduce the need for copying data.

#### 3.1.1 NDN-Based Solution

With NDN, data can come from anywhere, including in-network caches. Fast access to popular data reduces the need to download and store datasets locally. They can be quickly downloaded and deleted after the experiments. Multiple researchers in the same area can benefit from this approach since they no longer need to individually download datasets from NCBI, rather from a in-network cache that is automatically populated by the network. Further, the data downloaded from this cache can be verified publicly for provenance. Another solution is to push the computation to the data. This can be accomplished by adding a lambda (computational function) to the Interest name. The data source (e.g., a data producer or a dedicated service) interprets the lambda upon receiving the Interest and returns the computed results. Scientists don’t have to download and store large datasets every time they need to run an experiment.

### 3.2 Problem-Data Discovery

Genomics data is currently published from central locations (e.g., NCBI, NASA). The challenges in data discovery come not only from the fact that one needs to know all the locations of these datasets but also needs to navigate different naming schemes and discovery mechanisms provided by the hosting entity. There are many community-supported efforts to define controlled vocabularies and ontologies to help describe data [e.g. (Eilbeck et al., 2005; Schriml et al., 2012; The Gene Ontology Consortium, 2015)]. These metadata then can be parsed, indexed, and organized for data discovery. A scientist, for example, can associate appropriate metadata with source data, resulting data, and data collections. Moreover, the application of metadata to data is non-uniform, non-standard, and often inconsistent, making them difficult to utilize for consistent naming or data discovery.

#### 3.2.1 NDN-Based Solution

NDN does not provide data discovery by itself. Once data is named consistently by a certain community or subcommunity, these names can be indexed by a separate application (see our previous work (Fan et al., 2015) that provides name discovery functions and operated over NDN). Since name discovery in NDN is sufficient for data retrieval—an application can request for this name—no additional steps are necessary. Note that NDN only requires a hierarchical naming structure—how individual communities name their datasets (/biology/genome vs. /genome/biology) is up to them (Shannigrahi et al., 2020)

A distributed catalog (Fan et al., 2015) that stores the content names is sufficient to provide efficient name discovery. Since an NDN based catalog will only hold a community-specific set of names (not the actual data), the synchronization, update, and delete operations are lightweight (Shannigrahi, 2019). These names in these catalogs can be added, updated, and deleted as necessary. We refer the reader to our previous work for the details of how such a catalog can be created and maintained in NDN Fan et al., (2015).

### 3.3 Problem-Fast and Scalable Data Access

Currently, genomics data retrievals range from downloading a significant amount of data from a central data archive (e.g. NCBI) to downloading only the desired data that can be staged on local or cloud storage systems. For example, the researchers often need to retrieve genome reference data on demand for comparison. Downloading large amounts of datasets over long-distance Internet links can be slow and error-prone. Further, the current Internet model does not work very well over long distance links (tcp, 2020). Even with very high-speed links, it is particularly difficult to utilize all the available bandwidth.

#### 3.3.1 NDN-Based Solution

NDN provides access to data from “anywhere”, including storage nodes, in-network caches, and any entities that might have the data. This property allows scientists to reuse already downloaded datasets that are nearby (e.g., dataset downloaded by another scientist in the same lab). Additionally, in NDN data follows the content popularity, as it is cached in the in-network devices automatically. The more popular content is, the higher the likelihood it would be cached nearby. All data is digitally signed, ensuring provenance is preserved.

Getting content fast and from nearby locations may be convenient to download data when needed and delete them when the computation is finished. For example, the reference human genome has been downloaded by us and our students hundreds of times in the last 2 decades. Secure and verifiable data downloaded on demand will reduce the amount of storage needed.

### 3.4 Problem-Minimizing Transfer Volume

The massive data volume needed by genomic workflows can easily saturate institutional networks. For example, the size of sequence data (in FASTQ format) or processed sequence alignments (in binary sequence alignment map (BAM) format) for one experiment, can easily aggregate into terabytes. If stored in online repositories, these data might be downloaded many times by researchers extending existing studies, leading to high bandwidth usage. One solution is to subset the data and download only the necessary portion. However, several challenges remain—if multiple copies of the file exist, the network/application layers can not take advantage of that to pull different subsets in parallel.

However, depending on the size and type of the analysis being performed, subsetting of the data may not be appropriate. Currently, that means the scientist would be required to download all datasets (or staged at a remote site) before the computation can begin. However, instead of downloading large amounts of data, pushing computation to data might be much more lightweight.

For example, to determine if a scientific avenue (e.g., a large scale experiment with millions of genomes) or dataset is worth pursuing, the scientists often run smaller scale experiments for early signs of interesting properties. A key issue is determining the smallest number of records required to produce the same scientific result as the full dataset, and we previously point to a simple saturation point as determined by transcript detection (Mills et al., 2019). Once a saturation point has been reached, one could pause and examine the results. If there is an interesting signal, then there is nothing preventing the user from processing more sequence records. However, if there is no signal, one could drop the experiment and move on to other datasets. However, this method currently requires downloading the full experimental datasets and running computations against them.

#### 3.4.1 NDN-Based Solution

NDN supports subsetting the data at the source, and transferring only the necessary portions reduces bandwidth consumption. This is already possible through BAM file slicing to select data specific to genomic regions. With NDN, the request can carry the required subsetting parameters and allow the user/applications to download only the part of the data required for computation. NDN can also parallelize subsetting in the event that multiple slices are needed, and data is replicated over multiple repositories. When subsetting is not appropriate, NDN is able to push computation to data by appending the computation to the Interest name (or adding them as the payload to the Interest). The result comes back to the requester under the same name and is also cached for future use, reducing bandwidth usage. Furthermore, in some genomics workflows, caching of computation can reduce the load on the compute and servers (such as those hosted in NCBI or cloud platforms).

### 3.5 Problem-Secure Collaboration

Genomics data, especially unpublished or identifiable human data can be very sensitive. Scientists often need to secure data due to privacy requirements, non-disclosure agreements, or legal restrictions (e.g., HIPPA). Without a security framework, securing data and enforcing permissions becomes difficult. Suitable data access methods with proper access control is therefore required for privacy and legal requirements. At the same time, scientific collaborations often need to share data between groups without violating security restrictions. Suitable frameworks must exist for utilizing open source sequenced data for research, albeit with appropriately restricted access. The lack of an infrastructure that allows secure access to a large number of sequenced human genomes prevents population genetics researchers from identifying rare mutations or test hypotheses on analogous experiments which can lead to medical advancement. Encryption and data security models, along with proper access control is highly necessary as data breaches of protected data can lead to massive fines, forcing institutions to severely limit the scope of allowable controlled data access on local cyberinfrastructure.

#### 3.5.1 NDN-Based Solution

To support secure data sharing among collaborators, all data in NDN is digitally signed, providing data provenance. When privacy is needed, NDN allows encryption of content, facilitating secure collaborations. Furthermore, the verification of trust and associated operations (such as decryption) can be automated in NDN—this is called schematized trust (Yu et al., 2015). One example of schematized trust might be the following: a scientist attempting to decrypt a data packet starting with “/NCBI” must also present a key that is signed by “/NCBI” and begins with the “/NCBI/scientistA”. More complex, name-based trust schemes are also possible.

This section discussed NDN properties that can address data management and cyberinfrastructure challenges faced by the genomics community. In the following section, we present a pilot study that uses a current genomics workflow that demonstrates some of these improvements in a real-world scenario.

## 4 Method

**FIGURE 2:**
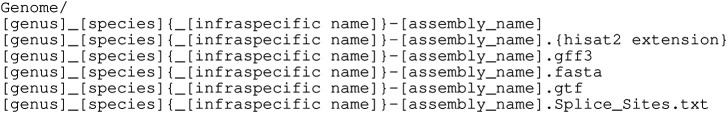
Genome naming strategy for indexing in NDN. The NDN names were translated from the existing Pynome file naming scheme. Tokens above (surrounded by square brackets) indicate the location of taxonomic names and genome assembly names. Most of the names directly map to hierarchical NDN names. Depending on the use case, components can be added or removed. These names are starting points for all NDN based operations. They are also the only necessary component for an NDN network.

**FIGURE 3:**
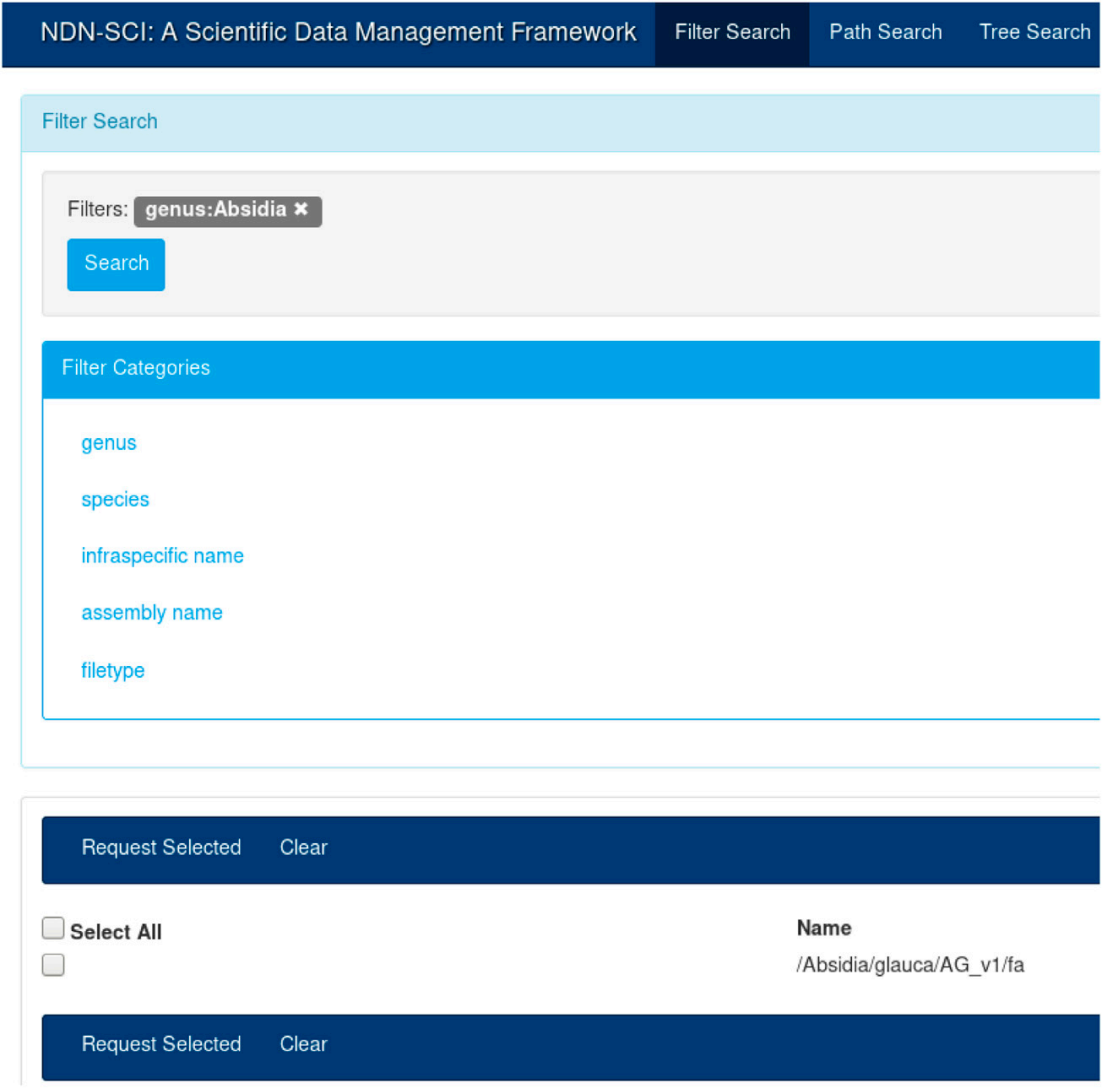
The user interface (UI) for the NDN-based genomics catalog system. It demonstrates how a name based catalog can act as a central point to an NDN ecosystem. Once the user looks up the names, additional functionality such as data retrieval can be built on top these names.

To demonstrate how NDN can benefit genomics workflows, we integrated NDN with a current genomics workflow (GEMmaker) and deployed our integrated solution over a real, distributed NDN testbed (Shannigrahi et al., 2015). The experiment has multiple parts: 1) naming data in a way that is understood by NDN as well as acceptable to the genomics community (Figure 2); 2) publishing data into the testbed and making them discoverable to the users using a distributed catalog and a UI (Figure 3); 3) Modify GEMmaker to interact with the data published in the testbed 4) Compare the performance of the new integration to the existing workflow. The following sections describe these efforts in detail.

### 4.1 NDN Testbed

For this work, we utilized a geographically distributed six-node testbed that was deployed at Colorado State University, ESNet, and UCAR supercomputing center. The testbed had six high-performance nodes (each with 40 cores, 128 GB memory, and 50 TB storage) and connected over ESnet (esn, 2020) using 10 Gbps dedicated links. All nodes ran the latest version of Fedora, and the network stack was tuned for big-data transfers (tcp, 2020). Specifically, we tuned the network interfaces to increase the buffer size and used large ethernet frames (9000-byte jumbo frames). We also tuned the TCP stack according to the ESnet specification, including increasing read and write buffers, utilizing cubic and htcp congestion control algorithms. We also tuned the UDP stack to increase read/write buffers as well as specifying CPU cores (tcp, 2020).

### 4.2 Data Naming and Publication

NDN recommends globally unique, hierarchical, and semantically meaningful names. This is a natural fit for the genomics community since they have established taxonomies dating as far back as 1773.

Note that while NDN requires names to be globally unique, there is no need for a global convention even among a particular community. For example, two names pointing to the same content, /Biology/Genome/Homo/Sapiens and /NCBI/Genome/Homo/Sapiens are perfectly acceptable. Each community is free to name their own content as they see fit. The uniqueness in these names come from the first component of the name (the prefix), which is /Biology and /NCBI, respectively. We anticipate each entity (e.g., an organization such as NCBI, a university, a community genome project) will have their own namespaces, possibly procured from commercial name registrars, the same way DNS namespaces are obtained today.

In the genomics community, very commonly used datasets can even be assigned their own namespaces. For example, a globally unique namespace (e.g., /human/genome) may be reserved for human genomes for convenience. However, that special namespace does not preclude an organization from publishing the same genomes from another namespace (e.g., /NCBI/human/genome). While NDN operates on names, it does not interpret the semantic meaning of the name components at the network layer. For example, NCBI announces the /NCBI prefix into the network that the NDN routers store. When an Interest named /NCBI/Genome/Homo/Sapiens arrives at the router, the router performs a longest prefix match on the name and matches the interface corresponding to /NCBI. This way, the network is able to forward Interests and data but does not need to interpret the individual components.

It is true that a name component (e.g., “hg38”) might have different meaning in different communities. It is the job of the application layer to interpret and use this components as they see fit. In our previous work, we built a catalog (an application) that mapped individual components to their semantic meaning (see Figure 3). Different communities will build different applications on top of NDN to understand the semantic meaning of a name.

The other problem is name assignment and reconciliation. In today’s Internet ICCAN (ica, 2020) and the domain registrars play a significant role in assigning, maintaining and reconciling DNS namespaces (e.g., assigning google.com namespace to Google). In NDN, these organizations will continue to control and assign namespaces that are used over the Internet.

In NDN, the data producer is responsible for publishing content under a name prefix (/biology or /NCBI). NCBI will acquire such namespaces from a name registrar. At that point, only NCBI is allowed to announce the /NCBI prefix into the Internet and publish content under that name prefix. The onus of updating namespaces is on the data publisher and the name registrars. Note that no such NDN name registrar exists today but we expect similar organizations to exist in the future.

When a user looks up a content name (e.g., /NCBI/Genome/Homo/Sapiens) in a catalog and expresses an Interest, the Interest is forwarded by the network and eventually reaches the NCBI server that announced /NCBI. Namespace reconciliation is not necessary for the network to function properly. Let’s imagine NCBI is authorized to publish datasets under two namespaces /Biology and /NCBI and it publishes the human genome under “/Biology/Genome/Homo/Sapiens” and “/NCBI/Genome/Homo/Sapiens”. A user can utilize both names to retrieve content—the network does not need to interpret the meanings of the names—the interpretation of name and content is up to the applications (and users) requesting and serving the datasets.

As part of the NSF SciDAS project (Sci, 2020), we store large amounts of whole genome reference and auxiliary data in a distributed data grid (iRODS). These whole genome references were initially retrieved from the Ensembl database (Zerbino, 2018) using the Python-based Pynome (Pyn, 2020) package and consist of hundreds of Eukaryotic species. Pynome processes these data to provide indexed files which are not available on Ensembl and which are needed for common genomic applications performed by researchers around the world. These data are organized in an evolution-based, hierarchical manner which is an excellent naming convention for an NDN framework.

For this study, we used an NDN name translator that we created as part of our previous work (Olschanowsky et al., 2014) to translate these existing names into NDN compatible names. Once translated, the content names became the unique reference for these datasets. Figure 2 shows the reference genome DNA sequence names created from the existing hierarchical naming convention. For example, one such name would look like “/Absidia/glauca/AG_v1/fa”. We could find that some of these names may or may not contain certain components, for example, infraspecific name in the above example. We then translated and imported these names into our NDN data management framework (Shannigrahi et al., 2018b). All subsequent operations such as discovery, retrieval, and integration with workflows used these names.

After naming, we published these datasets under the derived names on the NDN testbed servers. In this pilot test, we used three nodes to publish the data. However, each testbed server published the same data under the same name, replicating the content. Depending on the client location, requests were routed to the closest replica. If one replica went down, nothing needed to change on the client’s end—the NDN network routed the request to a different replica. We then used this testbed setup in conjunction with GEMmaker to test NDN’s usefulness. We discuss the results in the evaluation section.

The UI in Figure 3 provides an intuitive way to search for names that were published on the testbed. A user could create a query by selecting different components from the left-hand menu (e.g., by selecting “Absidia” under genus). The user can start typing the full name, and the UI will provide a set of autocompleted names. Finally, the user could choose to view the entire name tree using the tree view. The catalog and the UI are necessary for dataset discovery—while NDN operates on names, it is more efficient and fast to discover names using a catalog. Once discovered, the names could be used for all subsequent operations. For example, once the names are known, the user can initiate retrieval or other operations, as discussed before. The API also provides a command-line interface (CLI) for name discovery, allowing the users to integrate the name discovery (and subsequent operations) with domain workflows.

Since NDN operates on names, data naming in NDN affects application and network behaviors. The way a piece of content is named has profound impacts on content discovery, routing of user requests, data retrieval, and security. Besides, the naming of individual pieces of content seriously affects how the behaves. For brevity, we do not discuss those naming trade-offs here but point the reader to our recent work on content naming in NDN (Shannigrahi et al., 2020).

### 4.3 Integration With GEMmaker

GEMmaker (Hadish et al., 2020) is a workflow that takes as input a number of large RNA sequence (RNA-seq) data files and a whole genome reference file to quantify gene expression-levels underlying a specific set of experimental parameters. It produces a Gene Expression Matrix (GEM), which is a data structure that can be used to compare gene expression across the input RNA-seq samples. GEMs are commonly used in genomics and have been instrumental in several studies (Ficklin et al., 2017; Roche et al., 2017; Dunwoodie et al., 2018; Poehlman et al., 2019). GEMmaker consists of five major steps: 1) read RNA-seq data files as input; 2) trim low-quality portions of the sequences; 3) map sequences to a reference genome; 4) count levels of gene expression in each RNA-seq sample; 5) merge the gene expression values of each sample into a Gene Expression Matrix. GEMmaker is regulated by the Nextflow (NextFlow, 2019) (Di Tommaso et al., 2017) workflow manager, which automates the fluid execution of each step in GEMmaker, which are defined as Nextflow processes. Nextflow has a variety of executors that enable the deployment of processes to a number of HPC and cloud environments, including Kubernetes (K8s).

Without NDN, the typical process a user follows to execute GEMmaker in a K8s environment is as follows. First, the user moves the whole genome reference files onto a Persistent Volume Claim (PVC), a K8s artifact that provides persistent storage to users. Nextflow is able to mount and unmount GEMmaker pods directly to this PVC, which stores all input, intermediate, and output data. When the user executes GEMmaker, Nextflow deploys pods that automatically retrieve the RNA-seq data directly from NCBI using Aspera or FTP services. One pod is submitted for each RNA sample in parallel, so ideally all RNA-seq data is downloaded simultaneously. This represents the bulk of data movement in GEMmaker. Once each sample of RNA-seq data is downloaded to the PVC, new pods are deployed to execute the next step of the workflow for each sample. Each step in GEMmaker produces a new intermediate file used as input for the next step, all of which is written to the PVC. Once GEMmaker completes, the resulting GEM can be downloaded from the PVC by the user.

Currently, pulling data from host-specific services is built into the GEMmaker workflow. For example, the NCBI SRA-toolkit can pull data from SRA but not all genome data repositories. With NDN, the process can be abstracted from the workflow logic as data is preloaded into the NDN testbed from any host, downloaded by name at step 1, reference data is pulled by name at step 3, and data is created and uploaded with a new name at step 5. In contrast to the typical execution of GEMmaker, with NDN, users need not retrieve the whole genome reference or the final GEM, and retrieval of RNA-seq data can occur independent of Aspera or FTP protocols, supporting a greater variety of data repositories or even locally created data (so long as a local publisher makes it available). Users need only provide the NDN names for moving data and accessing cloud APIs.

For GEMmaker integration, we created a NDN-capable program that pulled data from the NDN testbed using the SRA ID. We modified GEMmaker to replace the existing RNA-seq data retrieval step with this NDN retrieval program and to retrieve the whole genome references. These whole genome references are the same described previously that were generated by Pynome and cataloged in the testbed. For this small-scale testing, we added the NDN routes to the testbed machines manually. However, the NDN community provides multiple routing protocols (Wang et al., 2013) that can automate the routing updates.

## 5 Results

### 5.1 Performance Evaluation

In order to understand how caching affects data distribution, we moved datasets of different sizes between the NDN testbed and Clemson University. The SRA sequences were published into the testbed, and then the workflow modified to download data over NDN. NDN based download only needs to know the name of the content. The rest of the infrastructure is opaque to the user.

For these experiments, the standard NDN tools for (Afanasyev et al., 2018) publication and retrieval were used. Once the files were downloaded, they were utilized in the workflow. For the first experiment, we copied different sized files using existing IP based tools (wget). For comparison, we then utilized NDN-based standard tools (ndncatchunks and ndnputchunks) for the same files, with in-network caching disabled followed by caching enabled. Each transfer experiment was repeated three times.

**FIGURE 4:**
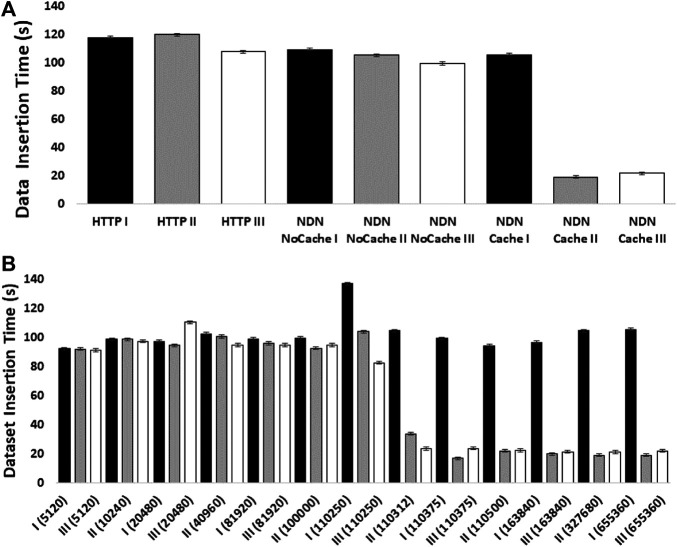
The effect of NDN caching on DNA dataset insertion into GEMmaker workflow. A set of three NCBI SRA *Arabidopsis thaliana* datasets (SRR5263229:167.9 Mb, SRR5263230:167.8 Mb, SRR5263231:167.9 Mb) was sequentially transferred three times (black bars (transfer I), gray bars (transfer II), white bars (transfer III)) for insertion into the GEMmaker workflow from the NDN testbed or from the SRA repository over the Internet via HTTP. The *y*-axis in both panels shows the transfer time of all three datasets from request to workflow accessibility in seconds. Error bars represent standard error of the mean. **(A)** The aggregate transfer times are shown for the three sequential transfers with HTTP, NDN tested without caching, and NDN with caching at 655,360 packets. **(B)** The effect of varying cache size (packet number in parentheses) is shown. The *x*-axis shows the cache capacity in packet numbers.

The experiments showed how NDN can improve content retrieval using in-network caching. Figure 4A shows a comparison of data download speed between NDN and HTTP. The first 3 bars represent three sequential downloads of a set of three SRA datasets from the NCBI repository using HTTP. The next 3 bars show three sequential downloads using NDN retrieval from the NDN testbed without caching. Since we manually staged the data, we knew that data source was approximately 1,500 miles away from the requester. Even then, the download performance was comparable with the HTTP performance. The real performance gain came from caching the datasets as seen in the last 3 bars where the first transfer was similar to HTTP and NDN without caching ( 2 min) while the next two transfers only took around 20 s after caching kicked in. These results point toward a massive improvement opportunity since many genomics workflows download hundreds or even thousands of SRAs for a given experiment.

Figure 4B shows how much in-network cache was needed to accomplish speedup using in-network caching and how the caching capacity affects the speedup. The *x*-axis of this figure shows the cache size in the number of NDN data packets (by default, each packet is 4,400 bytes). We find that at around 500 MB cache size, we start to see speed improvements.

**FIGURE 5:**
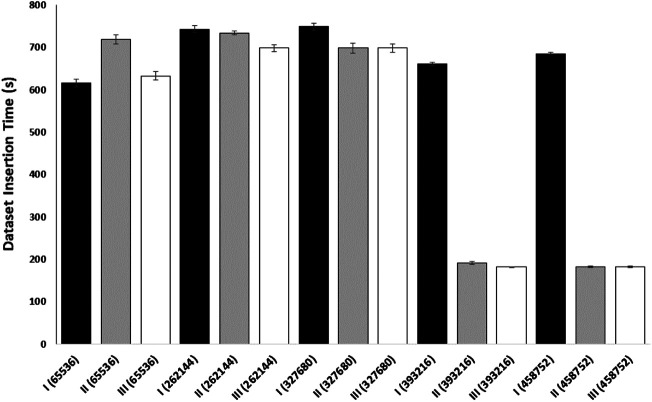
The effect of NDN caching on SRA download times on a Kubernetes cluster. A single NCBI SRA *Homo sapiens* kidney RNAseq dataset (SRR5139395) was downloaded a total of nine times (3 transfer trials labeled I, II, III in triplicate) for each cache size (packet number in parentheses). The following cs_max sizes (packet number) were used: 65,536 (approx 500 MB), 262,144, 327,680, 393,216, and 458,752. The *Y*-axis shows the download time for the dataset into the endpoint pod in seconds. Error bars represent standard error of the mean.

We performed an additional experiment to better evaluate caching on data transfer in a cloud environment (Figure 5). In this caching experiment, gateway and endpoint containers were employed to determine the time it takes to download a SRA sequence dataset. The gateway container used NDN to pull the SRA sequence from a remote NDN repository and create the network cache. The endpoint container (with a cache size of 0) acted as the consumer and created a face to the gateway used to pull the data. Both the endpoint and the client were run on separate nodes to replicate real cloud scenarios. This experiment demonstrates how an NDN container can cache a dataset for use by any endpoint on the same network and indicates that an insufficiently sized content store on the gateway will prevent network caching, resulting in slower download times. This provides a basic example of how popular genomic sequences could be cached for use by multiple researchers working on the same network in the cloud.

The actual cache sizes in the real-world would depend on request patterns as well as file sizes—we are currently working on quantifying this. In any case, it is certainly feasible to utilize NDN’s caching ability for very popular datasets, such as the human reference genomes. Our previous work shows that in big-science communities, even a small amount of cache significantly speeds up delivery due to the temporal locality of requests (Shannigrahi et al., 2017).

## 6 Codebase

All code used for these experiments are publicly available and distributed under open source licences (Table 1). NFD is the Named Data Networking forwarder that works as a software router for Interests and Data packets. ndn-cxx is an NDN library that provides the necessary API for creating NDN based “apps”. NDN catalog is an NDN based Name lookup system—an application can send an Interest to the catalog and receive the name of a dataset. The application can then utilize the name for all subsequent operations. Two versions of the catalog exists, ndn-atmos is written in C++ while ndn-python-catalog is implemented in python.

**TABLE 1:** Software packages used in this experiment.

Component	Purpose	Code	License
NFD	Named data networking software forwarder. Forwards NDN interest and data based on names	https://github.com/named-data/NFD	GPLv3, MIT, BSD, boost
Ndn-cxx	Named data networking application library	https://github.com/named-data/ndn-cxx/	GPLv3
NDN catalog	Catalog for NDN name lookup	https://github.com/named-data/ndn-atmos and https://github.com/satyaprakash-1729/ndn-python-catalog	GPLv2, Apache2.0
GEMmaker	A workflow for construction of gene expression count matrices (GEMs)	https://github.com/SystemsGenetics/GEMmaker	GPLv2
Nextflow	A bioinformatics workflow manager	https://github.com/nextflow-io/nextflow	Apache 2.0
Integration experiments	Modified GEMmaker workflow with NDN integration	https://github.com/mmcogle/GEMmakerCam	GPLv2
NDN tools image	NDN tools in a container	https://hub.docker.com/r/cbmckni/ndn-tools	GPLv3

The experiments were run on Linux machines (Fedora and Ubuntu). The machines were a mix of bare metal and virtual machines.

Nextflow is a general purpose workflow manager. GEMmaker is a genomics workflow adpated for Nextflow (see Section 4.3 above for more details). For these experiments, we modified GEMmaker to request data over NDN instead of standard mechanisms (such as HTTP). This modified workflow is available under this repository—https://github.com/mmcogle/GEMmakerCam.

## 7 Discussion and Future Directions

### 7.1 Discussion

This work demonstrates the preliminary integration of NDN with genomics workflows. While the work shows the promise of NDN toward a simplified but capable cyberinfrastructure, several other aspects remain to be addressed before NDN can completely integrate with genomics workflows and cloud computing platforms. In this section we discuss the technical challenges as well as the economic considerations that are yet to be addressed.

#### 7.1.1 Economic Considerations

NDN is a new Internet architecture that operates differently that the current Internet. Consequently, the users and the network operators need to consider the economic cost of moving to an NDN paradigm. This section outlines some of the economic considerations. There are two primary cost of moving to an NDN paradigm: the cost of upgrading current network equipment and the cost of storage if caching is desired.

As of writing this paper, NDN routers are predominately software based. To utilize this software based routers, the researcher needs to install NFD (an NDN packet forwarder) and NDN libraries on a machine (a server or even a laptop). All experiments in this paper were done on commodity hardware and did not require any additional capital investment. NDN is able to run as an overlay on top of the existing IP infrastructure or on Layer two circuits—in this work, we utilized NDN as an overlay on existing Internet connectivity.

Any commodity hardware (servers or desktop) with storage (depends on the workflow requirement) and a few GB memory is capable of supporting NDN. Given the current low cost of storage (an 8 TB hard drive costs around $150) even the cost of moving to a dedicated server is low. However, workflows with large storage requirement will need some capital investment. To minimize this cost and provide an alternate, we are working on two storage access mechanisms. First, when installing new storage is feasible (e.g. cost of storage continues to fall and a petabyte of storage costs around $30k at the time of writing this paper) it might be convenient for large research organizations to install dedicated storage that holds and serves NDN data packets—this approach improves NDN’s performance since objects are already packetized and signed by the data producers. The second approach is interfacing NDN with existing storage repositories such as HTTP and FTP servers. As NDN Interests come in, they are translated into appropriate system calls (e.g., POSIX, HTTP, or FTP) and the NDN Data packets are created on-demand. This approach is slower than the first approach but does not require any additional hardware or storage, reducing the deployment cost.

The benefits of NDN (caching etc.) becomes apparent when more users utilize an NDN based network. As the networking community moves toward testbeds and deployments such as NSF funded FABRIC (Dataset, 2020) that incorporate NDN into their core design, the research labs and institutes connected to these networks would be able to take advantage of those infrastructures. Additionally, connecting a software NDN router to these networks and testbeds are often free (assuming an institute is already paying for Internet access). These networks will create and deploy large scale in-network caches near the users and in the Internet core as users continue to request data from. As users exchange data over these networks, data will be automatically cached and served from in-network caches. In the future, when ISPs deploy NDN routers, the researchers will be able to take advantage of in-network caching without added cost. However, we expect large scale ISP deployment of NDN to take a few more years.

**FIGURE 6:**
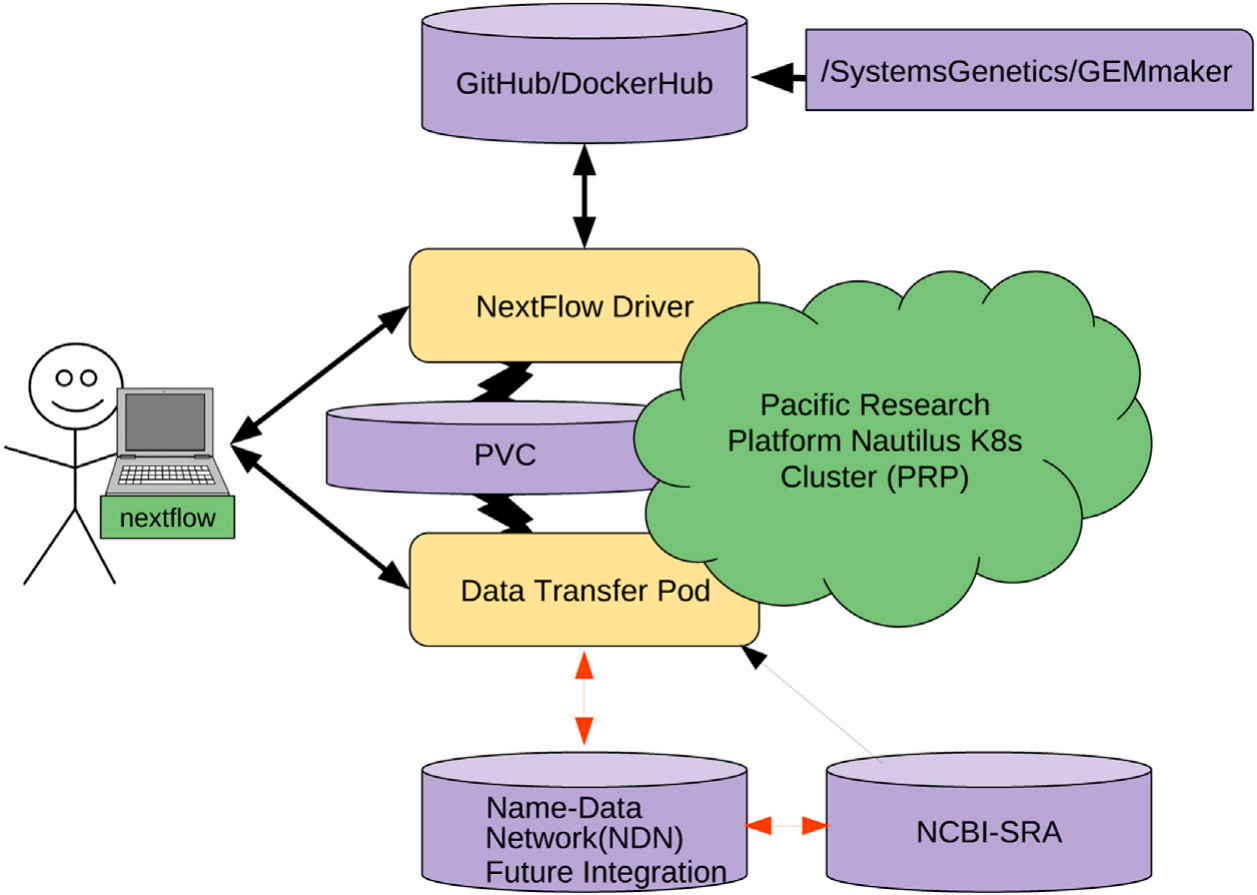
Nextflow managed GEMmaker workflow on a Kubernetes Cluster.

The other cost is the learning and integration cost with existing workflows. This is not trivial—NDN requires careful naming considerations, aligning workflows with a name base network, and data publication. To make this process straightforward, (Figure 6), we are working on containerizing several individual pieces. We hope that containerizing NDN, data transfer pods, and other components would allow the researchers to simply mix-and-match different containers and integrate them with workflows without resorting to complex configuration and integration efforts. Having discussed the economic considerations, we now discuss the technical challenges that remain.

### 7.2 Future Directions

#### 7.2.1 Software Performance

While our work shows some attractive properties of NDN for the genomics community, there are well known shortcomings of NDN. For example, the forwarder we used (NFD) was single threaded and therefore its throughput is low. This has been recently addressed by a new forwarder (ndn-dpdk) that can perform forwarding at 100 Gbps. We are currently working on integrating the genomics workflow with NDN-DPDK. We hope to demonstrate further improvements as the protocol and software stack continues to mature.

#### 7.2.2 Accessing Distributed Data Over NDN

Genomics data generation is highly distributed as data is generated in academic institutions, research labs, and the industry. While the HTDS datasets are eventually converted into in FASTQ format (Cock et al., 2010), the storage and downstream analysis data formats are highly heterogeneous with different naming systems, different storage techniques, and inconsistent and non-standard use of metadata. Seamlessly accessing these diverse datasets is an enormous challenge. Additionally, multiple types of repositories exist with different scopes—national-level (e.g., NCBI, EBI, and DDBJ) with large scale sequencing data for many organisms, community-supported repositories focused on a species or small clade of organisms, and single investigator web sites containing ad-hoc datasets. Storing these datasets in different repositories that are hosted under various domain names is unlikely to scale very well for two primary reasons—first, different hosting entities utilize different schemes for data access APIs (e.g. URLs) making it necessary to understand and parse various naming schemes, and second, it is hard to find and catalog each institutional repository.

NDN provides a scalable approach to publish and retrieve immutable content using their names in a location-agnostic fashion. NDN uses the content names for routing and request forwarding, ensuring all publicly available data can be access directly without the need for frequent housekeeping. For example, currently moving a repository under a new domain name requires a large amount of housekeeping, such as renaming the data under a new URL or linking new and old data. With NDN, the physical location of the data has no bearing on how they are named or distributed. When data is replicated, NDN brings the requests to the “nearest” data replica—“nearest” in NDN can be defined as the physically nearest replica, the most performant replica, or a combination of these (and other) factors.

However, several unexplored challenges exist on applying NDN to distributed data. First, NDN operates on names. Finding these names require the service of a third party software or name provider. A catalog that enumerates all names under a namespace (as we discuss before) can provide this service. However, it is not yet obvious who would be responsible for running and updating the authoritative versions of these catalogs. When data is replicated, we also need to address the issue of data consistency across multiple repositories. This is still an active research direction that requires considerable attention.

##### 7.2.3 Distributed Repositories Over NDN

A single centralized repository can become a bottleneck when subjected to a large number of queries or download requests. Moreover, it becomes a single point of failure and introduces increased latency for distant clients. NDN makes it easier to create distributed repositories since they no longer need to be tracked by their IP addresses. For example, in this work, we created a federation of three geographically distributed repositories. These repositories had to announce the prefix they intended to serve (e.g., “/genome”. Even when one or more repositories go down or become unreachable, no additional maintenance is necessary, NDN routes request to the available repositories as long as at least one repository is reachable. Similarly, when new repositories are added, the process is completely transparent to the user and does not require any action from the network administrator. However, several aspects remain to be addressed—how to data producers to publish long term data efficiently, how to replicate datasets across repositories (partially or completely), and how to retrieve content most efficiently.

#### 7.2.4 Publication of More Genomics Datasets and Metadata Into the NDN Testbed

There are currently hundreds of reference genomes in the NDN testbed and we are working on updating Pynome to include more current genome builds, genomes from services other than Ensembl, and support for popular sequence aligner programs (e.g., StAR, Salmon, Kallisto, and Hisat2). We are also working on loading the metadata and SRA RNA-seq files at scale into the NDN testbed. By studying the usage logs of our integration, we will better understand the benefit NDN brings to genomics workflows. Further, the convergence of searchable metadata from multiple data repositories published in the same NDN testbed will allow for a common search and access point for genomic data.

#### 7.2.5 Integration With Docker

For further simplification of workflows, we have created a docker container with NDN tools, forwarder, and application libraries built-in. The resulting container is fairly lightweight. We plan to publish the image to a public repository where scientists can download and utilize the docker build “as-is”. These images can be deployed to a variety of cloud platforms without modifications, further simplifying the NDN access to genomics workflows.

GEMmaker is able to run nextflow processes inside specified containers. By adding a container that is configured with NDN to the GEMmaker directory, scripts can run inside the NDN container during a normal GEMmaker workflow. The GEMmaker workflow can then use the ndn-tools to download the SRA sequences, both from ndn-only or NDN-interfaced existing repositories. This method also provides the opportunity for decreased data retrieval time due to NDN in-network caching and allows GEMMaker to benefit from all the NDN features we described earlier.

#### 7.2.6 Integration With Kubernetes

The genomics community is moving toward a cloud-based model. Container orchestration platforms (such as Kubernetes) are more commonly being used in favor of traditional HPC clusters. We believe that enabling users to easily move data from an NDN network to a Kubernetes cluster is imperative for the widespread adoption of this use case.

To achieve this goal, we are engineering the integration of NDN with a Data Transfer Pod (DTP) that is a deployment of different containers that enable users to read and write data to a Kubernetes cluster. A DTP is a configurable collection of containers, each representing a different data transfer protocol/interface. A DTP uses the officially maintained images of each protocol/interface, removing the need for integration aside from adding the container to the DTP deployment, which is an almost identical process for each protocol/interface. This makes adding new protocols very simple, as there is no need to build a custom image for each protocol/interface, as long as an image already exists. Almost all interfaces/protocols used by the community have officially maintained images associated with them.

The DTP tool will allow Kubernetes users to easily access data stored on NDN-based repositories. Each DTP container provides the client with a mechanism to utilize a different data transfer method (e.g. NDN, Aspera, Globus, S3). Using a DTP aims to address the need for simple access to data from a variety of sources. A DTP is not coupled with any particular workflow, so users will be able to pull or push NDN data as a pre- or post-processing step of their workflow, without modifying the workflow itself. The DTP can also be used to pull data from other sources if it is not present in an NDN framework.

We are modifying the existing GEMmaker workflow to use an NDN via the DTP that queries the NDN framework for the required reference genome files and SRA datasets. If the SRA dataset file exists in the NDN framework, the DTP will pull the data onto a Kubernetes persistent volume claim (PVC). For example, we are adapting NDN to work with the Pacific Research Platform (PRP; (Smarr, 2018)) Nautilus Kubernetes cluster (6). If the user knows the content name (or metadata) but content does not exist in NDN format, the DTP will pull the dataset from SRA with Aspera and publish it in the NDN testbed and then pull into PRP. Once published, the dataset will now exist in the NDN testbed and benefit from NDN attributes, including caching. Once the DTP completes its job, the GEMmaker workflow will function in the same way it does now so no new code needs to be written. We are also developing a cache retention policy to allow the SRA files to evaporate if they are not accessed after a certain period of time.

### 7.3 Limitations

The NDN prototype we used (NFD) and other components we used (catalogs and repo) are research prototypes. The performance and scalability of these prototypes are being improved by the NDN community. Additionally, utilization of NDN containers on PRP has not been explored before—we are working on optimizing both the containers and their interactions with the cloud platforms. We are also working on better understanding the caching and storage needs of the genomics community by looking at real-world request patterns and object sizes associated with them.

## 8 Conclusion

In this paper, we enumerate the cyberinfrastruture challenges faced by the genomics community. We discuss NDN, a novel but well-researched future Internet architecture that can address these challenges at the network layer. We present our efforts in integrating NDN with a genomics workflow, GEMmaker. We describe the creation of an NDN-complaint naming scheme that is also acceptable to the genomics community. We find that genomics names are already hierarchical and easily translated into NDN names. We publish the actual datasets into an NDN testbed and show that NDN can serve data from anywhere, simplifying data management. Finally, through the integration with GEMmaker, we show NDN’s in-network caching can speed up data retrieval and insertion into the workflow by six times.

## Data Availability

The datasets presented in this study can be found in online repositories. The names of the repository/repositories and accession number(s) can be found in the article/Supplementary Material.
